# Genome-wide analysis of WRKY transcription factors in *Aquilaria sinensis* (Lour.) Gilg

**DOI:** 10.1038/s41598-020-59597-w

**Published:** 2020-02-20

**Authors:** Yan-Hong Xu, Pei-Wen Sun, Xiao-Lin Tang, Zhi-Hui Gao, Zheng Zhang, Jian-He Wei

**Affiliations:** 1 Key Laboratory of Bioactive Substances and Resources Utilization of Chinese Herbal Medicine, Ministry of Education & National Engineering Laboratory for Breeding of Endangered Medicinal Materials, Institute of Medicinal Plant Development, Chinese Academy of Medical Sciences and Peking Union Medical College, Beijing, 100193 China; 2 Hainan Provincial Key Laboratory of Resources Conservation and Development of Southern Medicine & Key Laboratory of State Administration of Traditional Chinese Medicine for Agarwood Sustainable Utilization, Hainan Branch of the Institute of Medicinal Plant Development, Chinese Academy of Medical Sciences and Peking Union Medical College, Haikou, 570311 China

**Keywords:** Plant stress responses, Plant sciences

## Abstract

The WRKY proteins are a superfamily of transcription factor that regulate diverse developmental and physiological processes in plants. Completion of the whole-genome sequencing of *Aquilaria sinensis* allowed us to perform a genome-wide investigation for WRKY proteins. Here, we predicted 70 *WRKY* genes from the *A. sinensis* genome and undertaken a comprehensive bioinformatic analysis. Due to their diverse structural features, the 70 *AsWRKY* genes are classified into three main groups (group I–III), with five subgroups (IIa–IIe) in group II, except two belong to none of them. Distinct expression profiles of *AsWRKYs* with RNA sequencing data revealed their diverse expression patterns among different tissues and in the process of whole-tree-inducing agarwood formation. Based on the expression characteristics, we predict some *AsWRKYs* are pseudogenes, and some may be involved in the biosynthesis of agarwood sesquiterpenes as activators or repressors. Among the tested genes treated with MeJA and H_2_O_2_, most of them are induced by H_2_O_2_, but downregulated by MeJA, implying the complexity of their involvement in signal transduction regulation. Our results not only provide a basic platform for functional identification of WRKYs in *A. sinensis* but important clues for further analysis their regulation role in agarwood formation.

## Introduction

The WRKYs is one of the largest superfamily of transcription factors in higher plants^1^, which are characterized by their unique WRKYGQK motif at the N-end and the metal chelating zinc finger motif (CX_4–5_CX_22–23_HXH or CX_7_Cx_23_HXC) at the C-end^2,3^. They are divided into three subfamilies (Group I, Group II and Group III) according to the number of WRKY domains and category of zinc-finger^2^. Group I contains two WRKY domains and one C_2_H_2_ zinc finger structure; group II contains one WRKY domain and one C_2_H_2_ zinc finger structure; group III contains one WRKY domain and one C_2_HC zinc finger structure. Group II can be divided into five subgroups (IIa-IIe) according to the amino acid sequence. Studies have demonstrated that Group III only exists in higher plants, and most of them are related to plant response to biological stress, while Group I exists not only in higher plants, but also in ferns and some eukaryotic cells that can’t photosynthesize, such as myxomycetes and single-cell protozoa^1,4^. This suggests that WRKY transcription factor may originate from eukaryotic cells and Group I is the original form.

As transcription factor, WRKYs function by binding to specific sequences on the promoter of target genes. W-box, with consensus sequence (C/T)TGAC(T/C), is the specific recognition site of WRKYs. TGAC is its core conserved sequence, which is directly related to the specificity of WRKY transcription factor and its target downstream. One mutation will significantly reduce the binding activity and even completely disappeared^2,5,6^. Studies demonstrated that all WRKY proteins could bind to W-box except for SPF1^7^.

Numerous studies have demonstrated that the WRKY transcription factors play important roles in plant response to both biotic and abiotic stress^3,8–13^, especially in the control of plant disease and pathogen, it works as a core factor involved in plant defense response^12^. Many studies show that WRKY (e.g. AtWRKY22, AtWRKY29, AtWRKY33) is downstream of MAPK cascade^8,14–20^. It was found that, using pathogen infection or spraying SA on *Arabidopsis*, expression of 49 WRKY transcriptional factors out of the 74 were changed^8^, demonstrating the WRKY transcription factors are involved in many physiological process in plants. At present, 8 WRKY genes (*AtWRKY18/38/53/54/58/59/66/70*) have been confirmed directly downstream of NPR1^21^.

The WRKY transcription factors also play important roles in secondary metabolite biosynthesis including terpenes, flavonoids and alkaloids^22–25^. GaWRKY1 in *G. arboretum* participates in the biosynthesis of gossypol sesquiterpenes by regulating the activity of (+)-δ-Cadinene synthase (*CAD1*), and MeJA promoted this process^22^; AaWRKY1, a transcription factor isolated from glandular secretory trichomes in *A. annua*, binds the W-box of promoter and actives expression of Amorpha-4,11-diene synthase (*ADS*), a sesquiterpene cyclase that catalyzes the conversion of farnesyl diphosphate into amorpha-4,11-diene in the biosynthesis of the antimalarial artemisin^23^. Similarly, MeJA strongly induces the expression of AaWRKY1 and *ADS* in trichomes^23^. OsWRKY13 can induce the expression of *CHS* gene, which is related to flavonoid biosynthesis^24,25^. CrWRKY1 transcription factor regulates indole alkaloid biosynthesis by binding to the TDC gene W-box^26^; CjWRKY1, the first WRKY transcription factor identified in the biosynthetic pathway of alkaloids, regulates biosynthesis of Isoquinoline Alkaloid (IQA) in *Coptis jiponica*^27^. With the development of genome sequencing, medicinal plants have attracted much attention. Herein, an increasing number of WRKY transcription factors will be identified how they function in the regulation of secondary metabolism.

*Aquilaria sinensis* (Lour.) Gilg is the species of the genus *Aquilaria* of Thymelaeaceae. Agarwood, the resin portion that is widely used in traditional medicines, perfumes and incense across Middle East, Japan, India, China, and some Southeast Asian countries, is the product of its defensive response to external injuries^28–32^. We dedicated to the mechanism and technology of agarwood formation, and have completed genome sequencing of *A. sinensis*. To systematically elucidate the regulatory role of WRKY transcription factors in *A. sinensis*, in this study, a genome-wide analysis of AsWRKYs was performed. Totally, 70 *WRKY* transcription factor genes were predicted from *A. sinensis* genome, and 7 *AsWRKY* genes that likely regulate agarwood biosynthesis were identified based on their tissue expression patterns and the results of agarwit-treatment and qPCR analyses. Our results provide a foundation for understanding the molecular basis and regulatory mechanisms of WRKY transcription factors in *A. sinensis*.

## Results

### Identification of WRKY transcription factors in *A. sinensis*

The WRKYs are one of the largest families of plant transcription factors. The number of WRKY is different in different species. Among the lower plants, there are fewer WRKYs ranging from a few to dozens, for example, single in the unicellular *green alga*^1^ and 35 in *Spike moss Selaginella moellendorffii*^33^, while the higher plants ranging from dozens to hundreds, for example, 74 in *Arabidopsis*^1^ and almost 200 in soybean^34^. With the completion of genome sequencing, the number of WRKY transcription factors in many species has been revealed. Here, a total of 70 genes in the *A. sinensis* genome were identified as WRKY transcription factors that encoded 70 proteins. We named these members based on their order in the scaffold. Among these proteins, AsWRKY54 was identified to be the smallest protein with 115 amino acids (aa), whereas the largest one was AsWRKY66 (1505 aa). The molecular weights of the proteins also varied according to protein size ranging from 12.7 kDa (AsWRKY54) to 166.7 kDa (AsWRKY66).

### Gene structure, conserved motifs and chromosomal location of *AsWRKYs*

**Figure 1:**
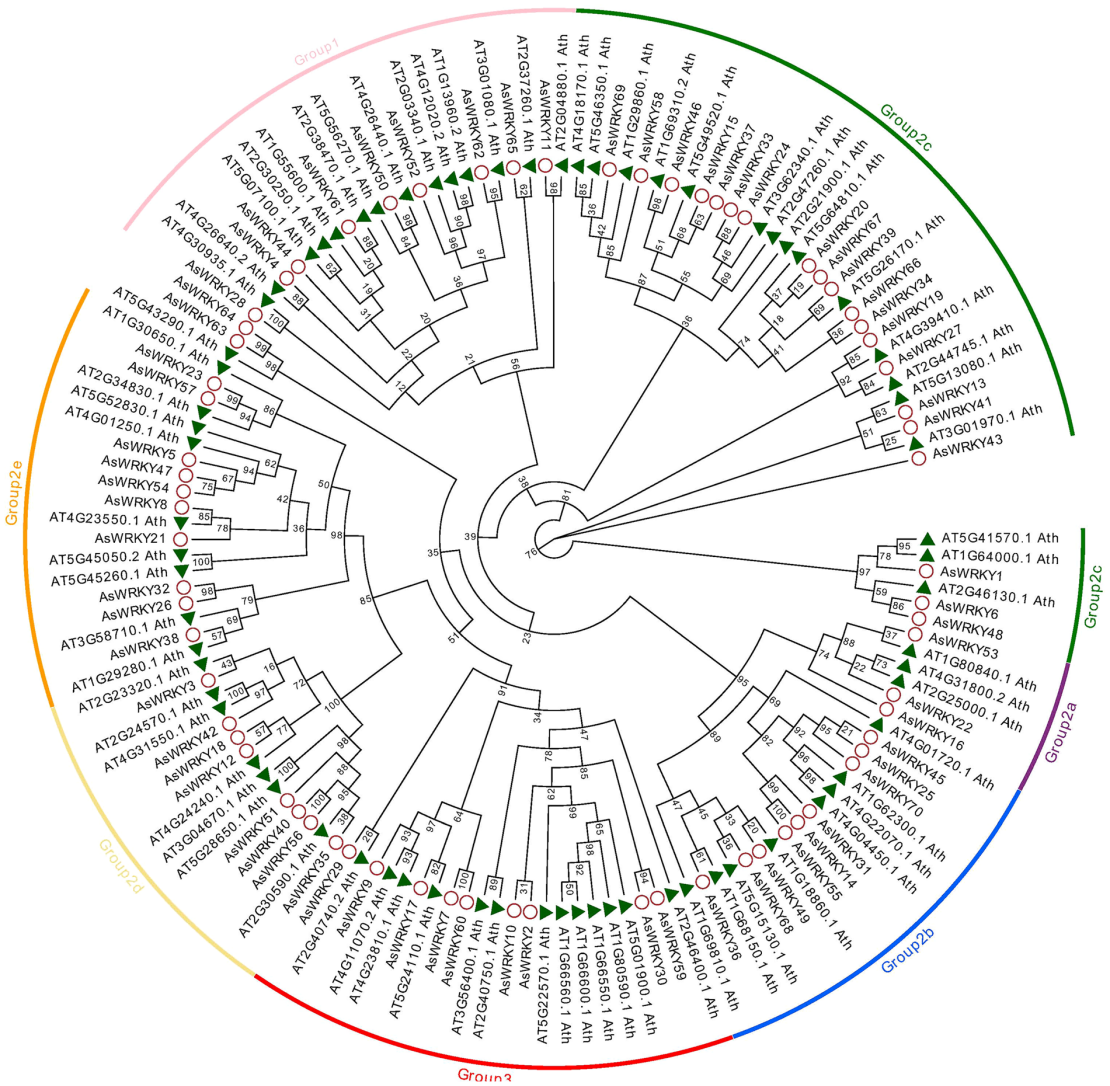
Polygenetic tree of AsWRKY proteins and homologous in *Arabidopsis*.

**Figure 2:**
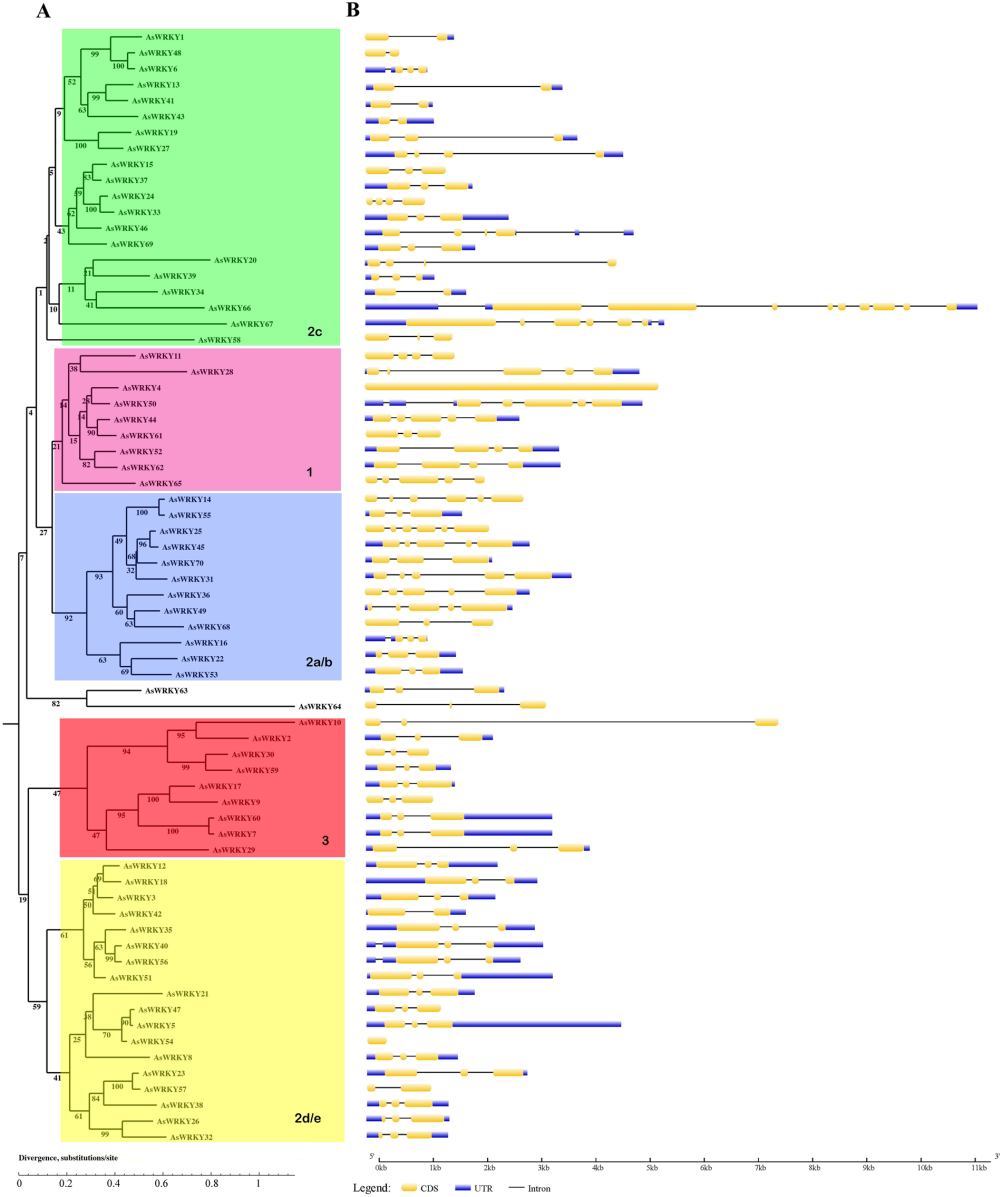
Phylogenetic tree and gene structure of *A. sinensis* *WRKY* genes. (**A**) The phylogenetic tree was constructed with MEGA 4.1 software by the neighbor-joining (NJ) method with 1,000 bootstrap replicates. (**B**) Exon/intron structure of *AsWRKY* genes: the introns, exons and UTR are represented by black lines, yellow boxes and blue boxes, respectively.

WRKYs are classified into three main groups typically according to their numbers of WRKY domains and zinc finger-like structures^35^. To look into the structural diversity of AsWRKYs, we first constructed a phylogenetic tree based on the full-length AsWRKY polypeptide sequences, and they were categorized into seven subfamilies as shown by Eulgem *et al*., 2000 (Fig. 1). Sequences from *Arabidopsis* WRKY members were included in our analysis as references. Of them, nine belong to group I, which have two WRKY domains (WRKYGQK) and two zinc-finger structure (CX_4_CX_22_HX_1_H, CX_4_CX_23_HX_1_H); nine belong to group III which have single WRKY domain (WRKYGQK) and one zinc-finger structure (CX_7_CX_23_HX_1_C); the other members are belong to groups II, and they have single WRKY domain. Though most of the sequences harbored the well conserved WRKYGQK motif and zinc finger motifs, variants were present in three of the groups II sequences, AsWRKY20, -67, -68, they each has 1 mismatched amino acid within the WRKY motif. The group II is sub-divided into 5 subgroup a-e. Specifically, AsWRKY63, AsWRKY64 were not assigned to any of the subgroup, which is consistent with the phylogenetic tree built on the basis of full-length AsWRKY genes using the neighbor-joining method in MEGA 4.0 (Fig. 2A). They are in the branch with one of the *Arabidopsis* At5G43290.1, which does not belong to any group in *Arabidopsis*, too^2^. This phenomenon is not special that exists in other plants. For example, among the 81 WRKYs in *Solanum lycopersicum*, 15 belong to group I, 52 belong to group II, 11 belongs to group III, and 3 belong to none of group^36^. The different WRKY subgroups from *A. sinensis*, *G. raimondii*, *Arabidopsis* and *S. lycopersicum* are described in Table 1.

**Table 1:** The number of the main three groups and their subgroups, as well as the total number of *WRKY* genes in *A. sinensis*, *G. raimondii* and *Arabidopsis*.

Group	subgroup	AsWRKYs	GaWRKYs	AtWRKYs	SiWRKYs
I		9	20	14	15
II	IIa	3	7	3	5
	IIb	9	16	8	8
	IIc	20	37	18	16
	IId	8	15	7	6
	IIe	10	13	1	17
III		9	12	13	11
none group		2		2	3
Total		70	120	74	

As gene structure is a typical imprint of evolution within a gene family, we analyzed the *WRKY* genes in *A. sinensis* using tools at the GSDS website. A detailed illustration of the gene structures is shown in Fig. 2B. The number of exons ranged from 1 to 10, and more than half of *AsWRKYs* have 3 exons and 2 introns. Each group showed similar structure, especially group III with group 2d/e (Fig. 2B), showing that almost all these members have tree exons and two introns. Most member in group I and group 2a/b contain three or four introns, while group 2c varies from one to nine. This phenomenon is also found in other plants, such as *populus*^37^, *soybean*^38^, and *setaria*^39^, demonstrating the conservation of the structure in WRKY family. It is also noted a few members, such as AsWRKY66, AsWRKY4 and AsWRKY54, showed quite different protein structures compared with other members. *AsWRKY66* has the largest numbers 10 exons and 9 introns. However, *AsWRKY4* and *AsWRKY54* have only 1 exon without containing any intron, and *AsWRKY4* is the longest exon. This loss of introns was considered as the result of intron turnover or due to reverse transcription of the mature mRNA followed by homologous recombination with intron-containing alleles^35,37^. It has been suggested that the transcription efficiency of genes may be related to the length of introns, and introns are relatively free to evolve faster than exons^40^. We also found that seven genes, including *AsWRKY10*, *AsWRKY13*, *AsWRKY19*, *AsWRKY20*, *AsWRKY27*,* AsWRKY28*, *AsWRKY29*, have longer intron than others, especially *AsWRKY10* has the longest intron more 6 kb (Fig. 2B), demonstrating mRNA transcripts would span over especially long intron for these genes.

**Figure 3:**
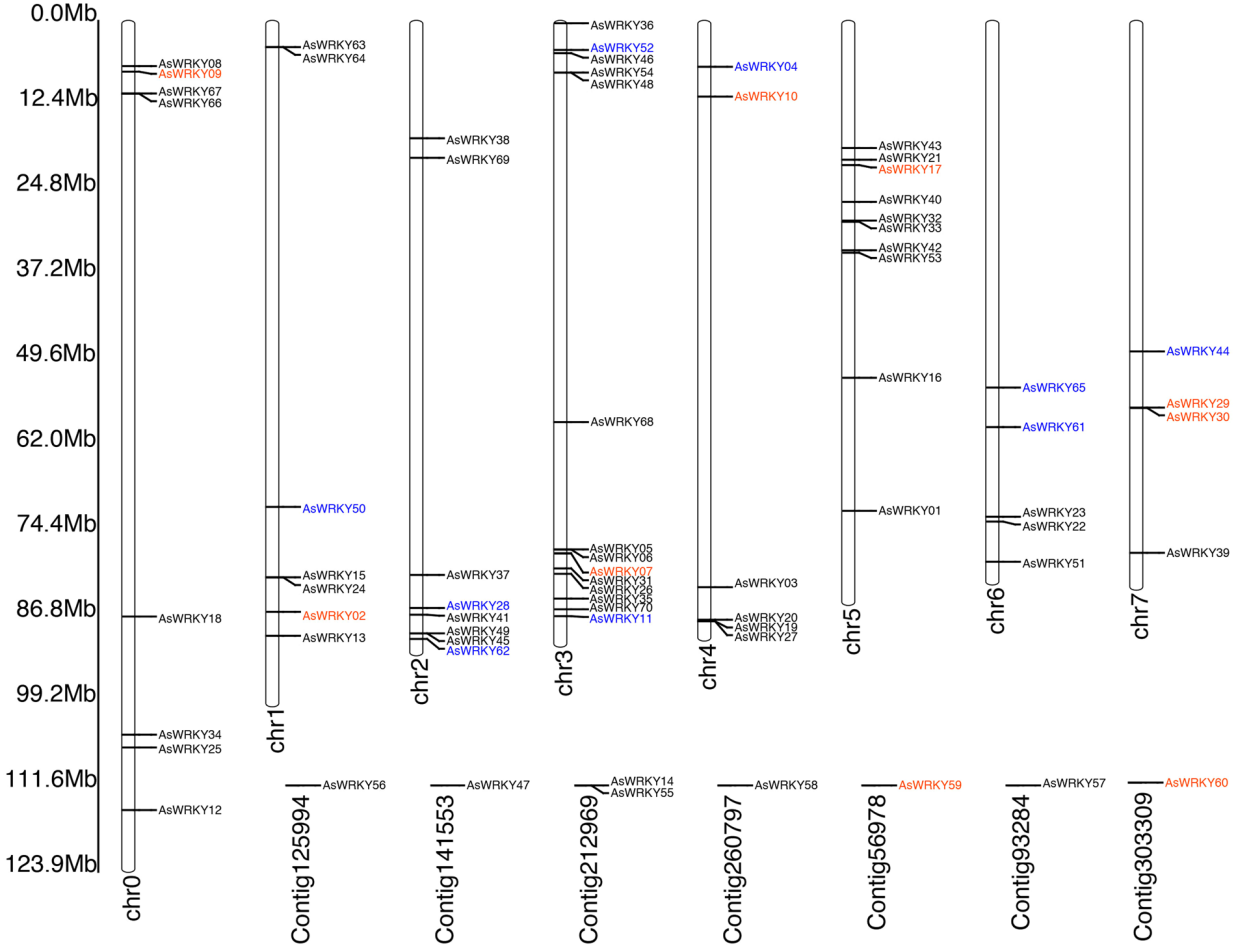
Distribution of *AsWRKY* genes on eight *A. sinensis* chromosomes. The chromosome number is indicated at the bottom of each chromosome. The genes marked in different color indicate the group of the WRKY family (I, blue; II, red; III, black).

The distribution of the *AsWRKY* genes on chromosomes was shown in Fig. 3. They were unevenly distributed throughout all eight chromosomes, and the number on each chromosome was not related to its length. Chromosome 3 (Chr 3) possessed the largest number of *WRKY* genes (14 genes) followed by Chr 5 (10 genes). Due to assembly of genome, 8 genes (*AsWRKY14/47/55/56/57/58/59/60*) could not be located on the chromosome.

### Tissue expression analysis of *AsWRKYs*

**Figure 4:**
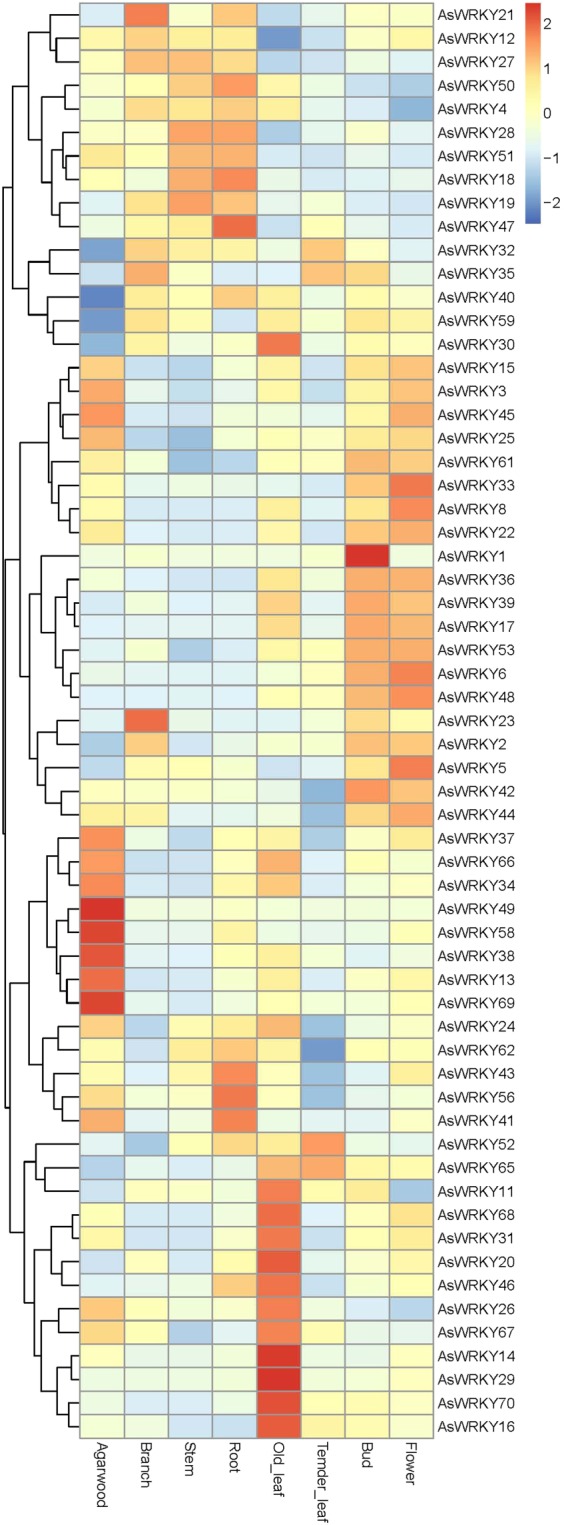
Heat map of the *AsWRKY* genes expression profiles in different tissues. All gene expression levels were transformed to scores ranging from −2 to 2 and were colored blue, white, or red to represent low, moderate, or high expression levels, respectively.

For the character that WRKY are widely involved in the developmental and physiological process of plant, expression profile of these *AsWRKYs* were analyzed from transcriptome data in 7 tissues including agarwood, root, branch, stem, old leaves, temder leaves, bud and flower. As shown in Fig. 4, the expression heatmap in different tissues are clustered. It is found that most of the *AsWRKYs* has a low expression level in temder leaves, some of them highly expressed in branch, stem and root, some expressed highly in bud and flower, some expressed highly only in old leaves, fully demonstrating the functional diversity of WRKYs. It is note that, 6 WRKYs (*AsWRKY7*,* -9*, *-10*, *-54*,* -63*, *-64*) were not expressed in none of the tissues, 18 were not expressed in agarwood, and 5 (*AsWRKY13*, *-38*, *-49*, *-58*, *-69*) were specifically highly expressed in agarwood, which is presumed closely related to the formation of agarwood. Interestingly, we also found that all of the* AsWRKYs* in group I expressed highly in all of the 7 tissues without obvious different expression level, which may indicate their fundamental roles in different cell-types in *A. sinensis*. All of group III has very low expression level in agarwood, branch, stem and root, even not expressed in agarwood. *AsWRKYs* expressed highly in agarwood belong to group II, correctly most of them (*AsWRKY13*, *-34*, *-37*, *-58*, *-66*, *-69*) belong to 2c, except to *AsWRKY49* belongs to 2b and *AsWRKY38* belongs to 2e. These results demonstrated that group III may not be involved in the agarwood formation, and the group II plays important regulatory role. It has been noted that gene expression patterns can provide important clues for gene function, these findings above may facilitate to identify the function of AsWRKYs and lead to discover their roles in plant growth and development, and in the process of agarwood formation as the biological functions of almost all of the AsWRKYs remain to be elucidated.

### Expression profiles of *AsWRKYs* in the process of whole-tree-inducing agarwood formation

**Figure 5:**
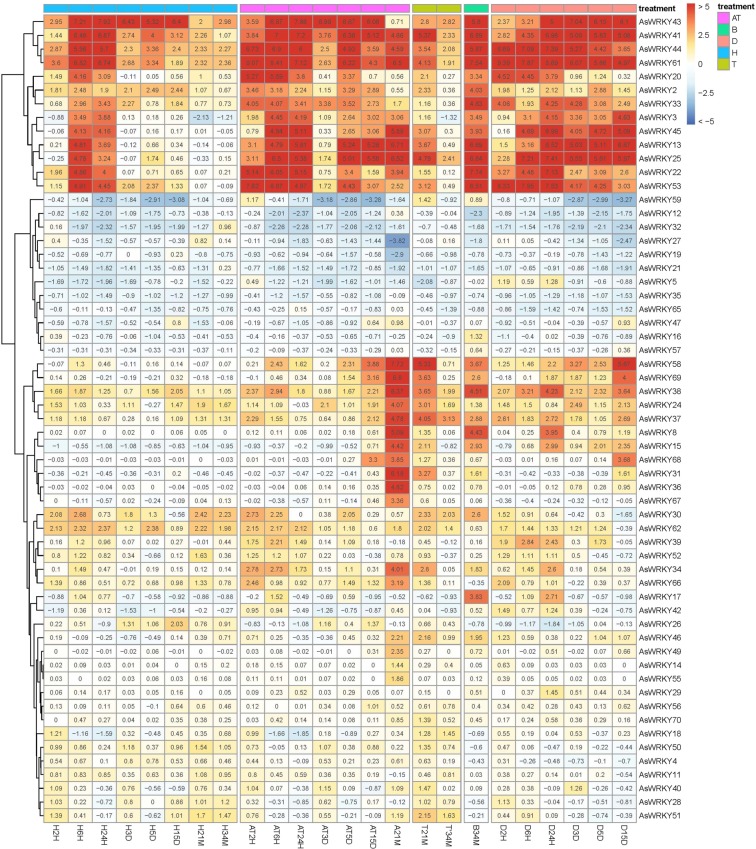
Heat map of the *AsWRKY* genes expression profiles in different layers and different agar-wit treated time. H- the healthy layer; D- the dead layer after Agar-Wit treatment, A- the agarwood layer; T- the transition layer; B- the blocked layer. All gene expression levels were transformed to scores ranging from −2 to 2 and were colored blue, white, or red to represent low, moderate, or high expression levels, respectively.

It has been demonstrated that WRKY TFs were involved in the activation of plant defense systems in response to stimuli^3^. Wound-induced agawood formation is the production of defense response in *A. sinensis*. To investigate whether AsWRKYs are involved in agarwood formation process, we analyzed the transcriptome data which performed using the whole-tree inducing materials, including different treatment-time and different layers. From the data and heat map (Fig. 5), it was found that there was a clear cluster, showing that some *WRKYs* highly expressed in all of the four layers (healthy layer—H, agarwood layer—AT and A, transition layer—T and decomposed layer–D), some *WRKYs* were highly expressed in agarwood layers, which we were concerned about. Of these different expression genes, some induced at the early stage (before 24 H), and some were induced later, especially only expressed in A21M, such as *AsWRKY49*, *AsWRKY68* and *AsWRKY69*. Combined with the tissue expression patterns, it was predicted that AsWRKY13, AsWRKY25, AsWRKY34, AsWRKY38, AsWRKY49, AsWRKY58, AsWRKY69 are likely important positive regulators in agarwood formation. Moreover, we also found some WRKYs (such as AsWRKY21, AsWRKY23) maybe the negative regulators in agarwood formation, for their expression were down-regulated after wound treatment. Moreover, some WRKYs could not be detected in all of layers, they are *AsWRKY7*,* AsWRKY9*, *AsWRKY10*, *AsWRKY54*, *AsWRKY63*, and  *AsWRKY64*. Combined with the tissue expression profile, the six genes were not be detected, and their RPKM values are zero. Herein, they are speculated to be pseudogenes.

### Investigation of *AsWRKYs* expressions in response to MeJA and H_2_O_2_ treatment exogenously by qRT-PCR

**Figure 6:**
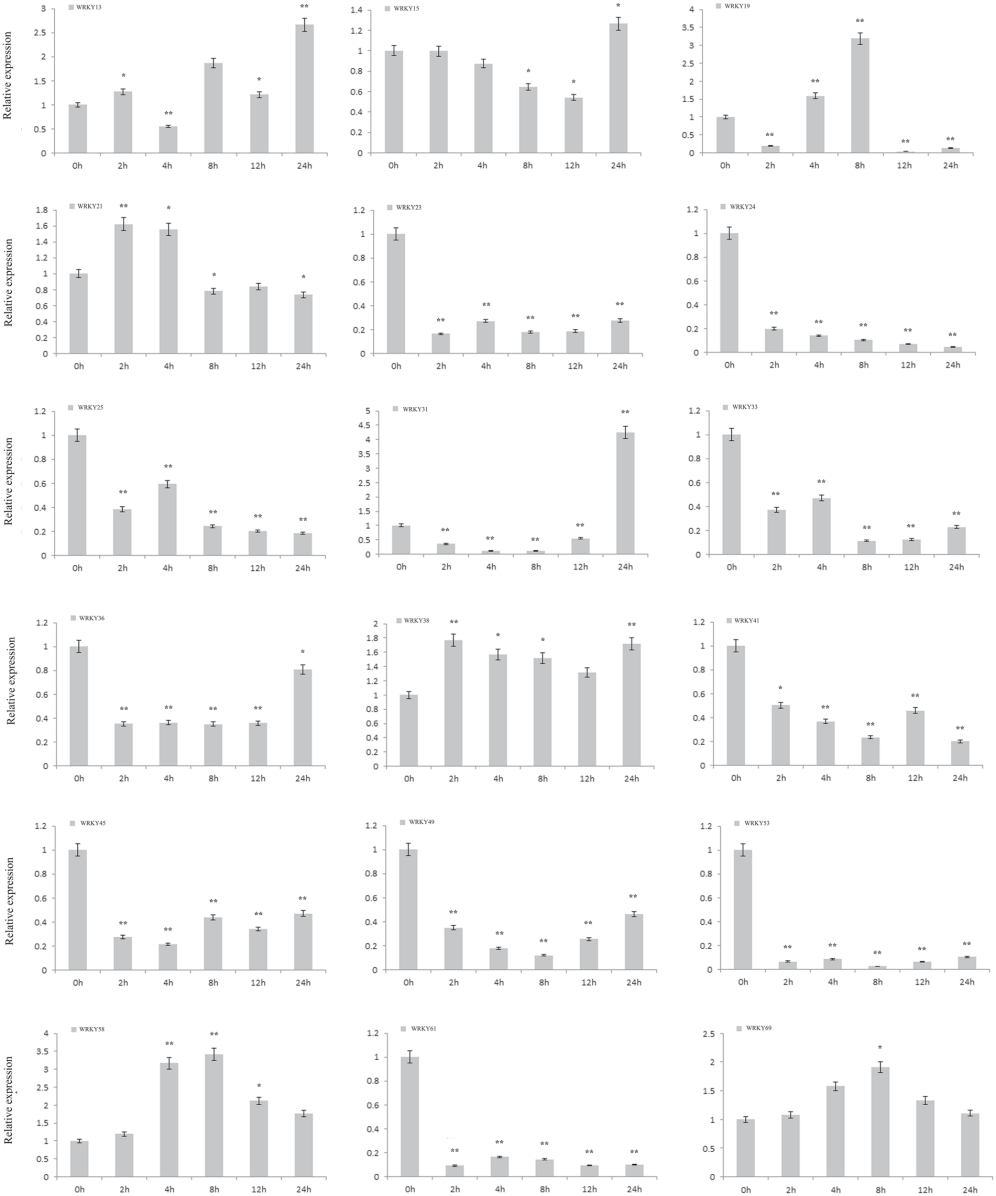
Expression profile of *AsWRKYs* in MeJA treated calli. *A. sinensis* calli were transferred to MS containing 100 μM MeJA and sample at appointed times (0 h, 0.5 h, 2 h, 4 h, 6 h, 12 h, 24 h). Expression levels of all detected genes were assayed using real-time PCR analysis and *AsGADPH* as the internal control. Each value is the mean ± SE of 3 independent biological replicates. Asterisks indicate significant differences between each treatment point and controls (0-h time point) (*P < 0.05, **P < 0.01, Student’s t-tests).

**Figure 7:**
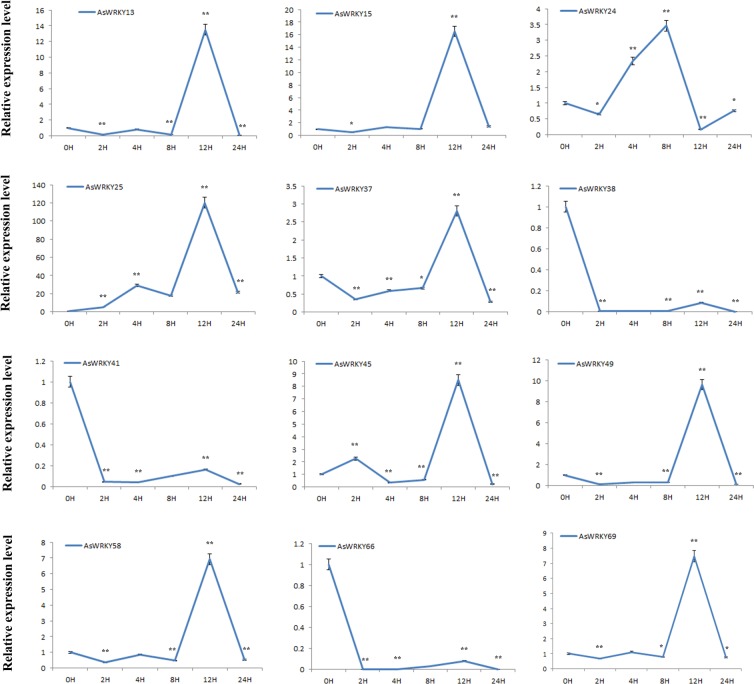
Expression profile of *AsWRKYs* in H_2_O_2_ treated calli. *A. sinensis* calli were transferred to MS containing 100 μM H_2_O_2_ and sample at appointed times (0 h, 0.5 h, 2 h, 4 h, 6 h, 12 h, 24 h). Expression levels of all detected genes were assayed using real-time PCR analysis and *AsGADPH* as the internal control. Each value is the mean ± SE of 3 independent biological replicates. Asterisks indicate significant differences between each treatment point and controls (0-h time point) (*P < 0.05, **P < 0.01, Student’s t-tests).

It has been confirmed that MeJA and H_2_O_2_ are important signal molecules involved in regulating agarwood formation^31,41^. To test the above expression analysis, we further detected the expression level of some genes responding to MeJA and H_2_O_2_ treatment by real-time PCR. They showed different characters in response to the two treatments. For MeJA treatment (Fig. 6), 6 were up-regulated and 9 down-regulated among the detected genes. Though the induction-fold was not obvious, the decrease rapidly and significantly. *AsWRKY23*, *AsWRKY24*, *AsWRKY53*, *AsWRKY61* declined 10 times rapidly within 2 hours and maintained stable until 24 h. *AsWRKY21* increased slightly and then decreased steadily, which may be a stress response to the external stimuli, and its regulation may be at the translational level. Expression of *AsWRKY15* and *AsWRKY38* were hardly affected, demonstrating they might not participate in the wound-induced agarwood formation. *AsWRKY38* and *AsWRKY69* are induced by MeJA and no decline within the 24 h, implying they are likely positive regulators. For H_2_O_2_ treatment by contrast (Fig. 7), most of detected *WRKYs* are induced by H_2_O_2_, except *AsWRKY38*, *AsWRKY41*, *AsWRKY66*, which dropped so sharply to their lowest level that barely detectable in two hours. Of the nine induced genes, *AsWRKY25* was found to be the strongest one with showing more 100 times, other eight peaked at 12 h, and then declined rapidly to the lowest level. In general, all the detected genes response more strongly to H_2_O_2_ than MeJA, which may consistent with our previous research result that hydrogen peroxide burst triggers accumulation of jasmonates in wound-induced agarwood formation^41^. These basic analyses provide important clues for further research.

## Discussion

WRKY proteins have been detected in various organisms^10^. Here, we systematically identified 70 WRKY members from whole genome sequences of *A. sinensis* for the first time. Sequence alignment and phylogenetic analysis classified them into three major groups (I, II, III) based on the WRKY domain and conserved zinc finger-like motif. Of them, group I and group III contains 9 members, respectively, and 50 belong to group II (Fig. 1) that is the largest group. However, in *Arabidopsis* and *poplar*, group I houses the largest number of WRKYs; while in rice, group III is the largest. The largest group in *A. sinensis* is group II, implying that this group may experience more gene duplications during the evolutionary course. At present, WRKYs found in the genomes of *Chlamydomonas monocytogenes*, non-photosynthetic eukaryotic *myxomycetes*, unicellular protoplasts, lower plant *bryophytes* and *ferns* belong to group I, demonstrating the group I is likely the original protein expression form and may originate from eukaryotic cells before undifferentiated plant kingdom 1.5–2 billion years ago^1^. Furthermore, by referring to the domain feature and the phylogenic tree (Fig. 2A), it showed three interesting observations: (i) all group I members have two WRKY domain and two zinc finger structure; (ii) AsWRKY20 possess only WRKY domain and lack zinc finger structure, and AsWRKY54 possess only zinc finger structure and lack WRKY domain; (iii) AsWRKY63 and AsWRKY64 belong to none of the group. All these results implied the AsWRKY genes may have experienced WRKY domain loss during the evolution.

In higher plants, the group III WRKY gene accounts for 20% of the family members, but it does not exist in some lower plants such as bryophytes. Studies have showed that almost all of group III WRKYs in *Arabidopsis* are related to biotic stress response^8^, which indicates that the group III WRKYs is relatively latest in the evolutionary history of terrestrial plants. However, in *A. sinensis*, all of group III has very low expression level in agarwood, branch, stem and root, even not expressed in agarwood (Fig. 4), and three of them (*AsWRKY7*, *-9*, *-10*) are considered as pseudogenes, implying the group III members may have little to do with the agarwood formation, which is inconsistent with the *Arabidopsis*.

Tissue expression analysis showed that group I expressed highly in all of the 7 tissues without obvious different expression level, all of group III has very low expression level in agarwood, branch, stem and root, even not expressed in agarwood. Members highly expressed specifically in agarwood belong to group II. Combined with their expression in the different layers of the agarwit treated, expression of members in group III did not change much during the whole process of agarwood formation, and only *AsWRKY61* in group I had highest expression in AT layer. So, AsWRKYs belonging to group II are predicted important regulators that act as activator or repressors of sesquiterpene biosynthesis. The results are consistent with their evolutionary classification that group I are the original proteins participating in various processes of growth and development, while group II has undergone great changes in the course of evolution. Anyhow, these results demonstrating that although WRKYs have conservative domains, their functions and regulations are very complex, and even the differences among members are very large.

There is a general consensus that agarwood can not be formed in healthy *A. sinensis* tree, only be induced after injury. In this study, we explored the possible role of WRKY in the formation of agarwood through tissue-specific expression and expression profiles in the process of agar-wit treated agarwood formation. From the tissue-expression heat map, it can be seen that *AsWRKY13/38/49/58/69* specific expressed in agarwood (Fig. 4), implying they may play a regulatory role in the formation of agarwood. WRKY proteins act as an activator or repressor regulating expression of downstream target genes in many different biological processes^14,42–44^. Some can function as activator in one pathway but as repressor in another^43^. The heat map in different layers and different agar-wit treated time showed that *AsWRKY13/25/34/38/49/58/69* may positive regulators, while *AsWRKY21/23* may function as negative regulators (Fig. 5). Of course, these information needs further verification. Our research group has previously confirmed that MeJA and H_2_O_2_ are important signal molecules involved in regulating agarwood formation^31,41^. Herein, we treated callus with MeJA and H_2_O_2_ and detected 15 genes expression of concern (Figs. 6, 7). It was showed that, among the 15 *AsWRKYs*, 9 are induced by H_2_O_2_, 3 induced by MeJA, and some genes showed an opposite response to MeJA and H_2_O_2_ treatment. Our previous study showed that hydrogen peroxide burst triggers accumulation of jasmonates in wound-induced agarwood formation^41^, the WRKY repressors and activators presented in the work will help test the model and contribute to the dissection of the transcriptional complex involved in MeJA and H_2_O_2_ signaling.

In summary, the WRKY family in *A. sinensis* was systematically identified, including their classification, chromosome distribution and their expression profiles in different tissues and response to different external stimulus, using comprehensive computational approaches and real-time PCR analysis. Our results could help to select appropriate candidate genes for further characterization of their functions in *A. sinensis*.

## Materials and Methods

### Plant materials for RNA-Seq

The materials for transcription sequence are seven-year-old *A. sinensis* stems grown in Hainan branch of IMPLAD (Institute of Medicinal Plant Development). Trees were treated using Whole-tree agarwood-inducing technique (Agar-Wit)^45^. Samples were collected at the pointed times. The healthy wood was used as control (0 h).

### Plant materials for qRT-PCR analysis

The materials for qRT-PCR are *A. sinensis* suspension. The suspensions have been cultured in dark. 100 μM MeJA or H_2_O_2_ was added to the suspension. Samples were taken at the pointed time after treatment. The suspensions without any treatment were used as control. All materials were frozen in liquid N_2_ and stored at −70 °C until used.

### Library preparation, transcriptome sequencing and bioinformatics analysis

Total RNA was isolated from the materials using a TRIzol kit (Invitrogen, USA) and purified to enrich messenger RNA (mRNA). Quantification and qualification RNA was checked to meet the standards for library construction. A total amount of 3 μg RNA per sample was used as input material for the RNA sample preparations. Sequencing libraries were generated using NEBNext^®^ Ultra™ RNA Library Prep Kit for Illumina^®^ (NEB, USA) following manufacturer’s recommendations and index codes were added to attribute sequences to each sample. The library preparations were sequenced on an Illumina Hiseq platform and 125 bp/150 bp paired-end reads were generated. Raw data (raw reads) were filtered to obtain the clean reads to ensure the quality of the information analysis. Index of the reference genome was built using Bowtie v2.2.3 and paired-end clean reads were aligned to the reference genome using TopHat v2.0.12.

### Identification of transcription factor of AsWRKYs and their chromosomal location

Transcription factors are characterized based on iTAK program (http://bioinfo.bti.cornell.edu/tool/itak)^46,47^. The program first compared species protein sequences against the pfam domain database^48^ using the HMMER3.1 program (http://hmmer.janelia.org). Meanwhile, GSDS software was used to map the *WRKY* gene structure. All parameters are default. *AsWRKY* genes were located on chromosomes according to their positions based on Hic (Chromosome conformation capture) result of *A. sinensis* genome.

### Phylogenetic and sequence feature analysis

WRKY protein blast was performed using the Muscle software, and then build a Neighbour-Joining tree using the TreeBeST of PAMLsoftware (bootstrap = 1000)^49^. The WRKY genes were classified based on their protein motifs and sequence similarity.

### Tissue expression and induced expression analysis

Calculate of the RPKM value of WRKY transcription factors in different tissues and log2(rpkm + 1) as the expression level, after normalization procedure draw the heat map. Similarly, heat maps at different time points in different layers were drawn according to the value of log2(rpkm + 1)/(H-0h -rpkm + 1). H-0h is the control. Here, the data was normalized using the Z-score standardization method (the mean value is 0 and the variance is 1). The expression level of each gene in each tissue (RPKM) was converted to Z fraction: Z_i_ = (X_i_-μ)/σ, Z_i_, X_i_, μ and σ represent Z fraction in i tissue, RPKM value and gene expression in i tissue, the mean value and the variance of gene expression.

### Expression profiling using qPCR

To analyze gene expression response to treatment of MeJA, and H_2_O_2_, exogenously, total RNA was isolated from treated cell suspensions using a Total RNA Rapid Extraction kit RN38-EASYspin Plus (Aidlab). 1-μg aliquot was subjected to first-strand synthesis according to the instructions of PrimeScript™ II 1st Strand cDNA Synthesis Kit (Takara). The primers used for real-time PCR are listed in Supplementary Table S1. Analysis was performed essentially according to our previously described procedures^31,50^.

## Supplementary information

Supplementary Table S1.
