# Supplementary material for: Genome-wide analysis of WRKY transcription factors in Aquilaria sinensis (Lour.) Gilg
Source: Sci Rep. 2020 Feb 20;10:3018. doi: 10.1038/s41598-020-59597-w (PMC7033210; doi:10.1038/s41598-020-59597-w)
Supplement: Supplementary file 1 — Supplementary Table S1. [file 41598_2020_59597_MOESM1_ESM.docx]

**Genome-wide analysis of WRKY transcription factors in *Aquilaria sinensis* (Lour.) Gilg**

**Running title: WRKY transcription factors in *Aquilaria sinensis***

**Yan-Hong Xu^1,^*, Pei-Wen Sun^1,^*, Xiao-Lin Tang^1^, Zhi-Hui Gao^1^, Zheng Zhang^1^, Jian-He Wei^1,2,§^**

^1^Key Laboratory of Bioactive Substances and Resources Utilization of Chinese Herbal Medicine, Ministry of Education & National Engineering Laboratory for Breeding of Endangered Medicinal Materials, Institute of Medicinal Plant Development, Chinese Academy of Medical Sciences and Peking Union Medical College, Beijing 100193, China

^2^Hainan Provincial Key Laboratory of Resources Conservation and Development of Southern Medicine & Key Laboratory of State Administration of Traditional Chinese Medicine for Agarwood Sustainable Utilization, Hainan Branch of the Institute of Medicinal Plant Development, Chinese Academy of Medical Sciences and Peking Union Medical College, Haikou 570311, China )

*These authors contributed equally to this work.

^§^Corresponding author: E-mail, [wjianh@263.net](mailto:wjianh@263.net); Fax, 86 10 57833358

**Supplementary Data**

**Supplementary Table S1. Gene-specific primers for real-time PCR analysis used in this Study.**

*AsWRKY13:*

forward primer: 5’- CTGATAAGAGTAAGACGGGTGAAGG -3’

reverse primer: 5’- CCACCACCCCTTCGTCTTTG -3’

*AsWRKY15*:

forward primer: 5’- GGGATGTGGGGTGAAGAAGAG -3’

reverse primer: 5’- GAGGAGGAGTAGTTACGGGTTTCG -3’

*AsWRKY19:*

forward primer: 5’- CGGCAAGAGAAGCAGTAGGG -3’

reverse primer: 5’- GGTCCTCCGCTAATCTTTCCAC -3’

*AsWRKY21:*

forward primer: 5’- CACCTCACCCAAGGAACTACTACC -3’

reverse primer: 5’- GACGACGCTTCTCCTGTTCC -3’

*AsWRKY23:*

forward primer: 5’- GGTCATAACCTACACATCGGAGC -3’

reverse primer: 5’- CTCCTTATTCTGTGTGTCATCGGC -3’

*AsWRKY24:*

forward primer: 5’- TCTTTCAGCGACCCGAGCAT -3’

reverse primer: 5’- CTATTCCCAAAGTTCAAAGGCAAG -3’

*AsWRKY25:*

Forward primer: 5’-TGGGCTAATGAATCCAAATCTGC -3’

Reverse primer: 5’-CCGCCTACTTGTGACGACGC -3’

*AsWRKY31:*

Forward primer: 5’-CCCTCCCACTACCTCCCTCAC-3’

Reverse primer: 5’-GACCGTATCAATCAACGACGAGTG-3’

*AsWRKY33*:

Forward primer: 5’-TTATCGTTGCACTACTGCCTCATG-3’

Reverse primer: 5’-TGACACAGATGTTGGTTTATTTGC-3’

*AsWRKY36*:

Forward: primer: 5’-ACTGTCGCTCCAAGTTGTCCC-3’

Reverse primer: 5’-AGAAGTAGTGTTGGTGGGGACG-3’

*AsWRKY37*:

Forward primer: 5’-GACCATTTAGATGACGGCTACAGAT-3’

Reverse primer: 5’-GGACCACAAAAGAGGAAGCCC-3’

*AsWRKY38*:

Forward primer: 5’- CCGAGGTGGAGACTGAACCAGAGCC-3’

Reverse primer: 5’-GAAAAACATTCCCAAGTCAGCTC-3’

*AsWRKY41*:

Forward primer: 5’-AGCGAAAACCAAGAAGCATAACA-3’

Reverse primer: 5’-TGTCAGTAGGCTTCTCGATGGG-3’

*AsWRKY45*:

Forward primer: 5’-CCACCATTTCTGCTTCGGC-3’

Reverse primer: 5’-GATGGAGGAGATGGCGGCA-3’

*AsWRKY49*:

Forward primer: 5’-CACAACAGTCCTTGACAGAAACCTT-3’

Reverse primer: 5’-AGATAGAGAAAGGTGGCTGTGAATG-3’

*AsWRKY53*:

Forward primer: 5’-GTCACAAGAGATAACCCTTCACCC-3’

Reverse primer: 5’-TTTCTCGCCCGAATCCTCTG-3’

*AsWRKY58*:

Forward primer: 5’-CGCTGCCGAAAGTAATCCATT-3’

Reverse primer: 5’-ACTCTCTTCTTCACCGTGCACTTC-3’

*AsWRKY61*:

Forward primer: 5’-CTTCGGATTCGTTAGGTGAGGA-3’

Reverse primer: 5’-TCGGGTTTCCCTTCACTACTTTC-3’

*AsWRKY66*:

Forward primer: 5’- GGAGAGGGGAGAACAAGATGATG-3’

Reverse primer: 5’-AAGCCATCGTCAAGGACTTCAAC-3’

*AsWRKY69*:

Forward primer: 5’-GCCAGCACAACCACCCCA-3’

Reverse primer: 5’-TAAAAGTTCGGCGGGTAGATAGAG-3’
